# Effects of atmospheric oscillations on infectious diseases: the case of Chagas disease in Chile

**DOI:** 10.1590/0074-02760180569

**Published:** 2019-06-03

**Authors:** José C Báez, Jesús Olivero, Lorena E Salazar-Aravena, Iván C Suazo-Galdames

**Affiliations:** 1 Universidad Autónoma de Chile, Facultad de Ciencias de la Salud, Instituto de Ciencias Biomédicas, Santiago, Chile; 2 Instituto Español de Oceanografía, Centro Oceanográfico de Málaga, Málaga, Spain; 3 Universidad de Málaga, Facultad de Ciencias, Departamento de Biología Animal, Málaga, Spain

**Keywords:** atmospheric teleconnections, SOI, AAO, temporal predictions

## Abstract

**BACKGROUND:**

Currently, there is an increasing global interest for the study of how infectious diseases could be linked to climate and weather variability. The Chagas disease was described in 1909 by Carlos Chagas, and is caused by the flagellate protozoan *Trypanosoma cruzi*. The Chagas disease is considered one of the biggest concerns in public health in Latin America. In Chile, the main vectors involved in the transmission of *T. cruzi* are arthropods of the Triatominae subfamily. Moreover, another main transmission way is through of vectors by fecal-urine way, however, oral way also has been described among others transmission form.

**OBJECTIVES:**

In order to get understand outbreaks of Chagas-disease, we search for possible relationships between the frequency of cases in the Chilean population and atmospheric oscillations.

**METHODS:**

We explored the two most important atmospheric oscillations in the Southern Hemisphere: southern oscillation index (SOI) and Antarctic oscillation (AAO), during the available years with official data. Because the number of migrant people born outside from Chile increasing significantively between 2014 and 2018, we used for the analysis two different periods from data available official data: (i) 2001 to 2014, (ii) 2001 to 2017.

**FINDINGS:**

For both periods we observed a significant and positive relation between AAO one year before. However, for the 2001 to 2014 period positive SOI one year before, which is related with La Niña phases, was the more important variable.

**MAIN CONCLUSIONS:**

The Chagas disease frequency per year in Chile was found to depend mainly on SOI in previous year, whose values can be determined one year in advance. Therefore, it is possible to partially forecast annual frequency patterns. This could have important applications in public health strategies and for allocating resources for the management of the disease.

The world is currently experiencing a period of rapid global warming,^1^ primarily driven by human activity.^2^ Although there is an increasing concern over the impact of global warming on human health, such as food safety,^3^ it is difficult to predict its influence in public health. In this context, climate change is expected to increase the prevalence of a wide range of health risks, mainly those derived from insect transmission such as Malaria,^4^ ^,^ ^5^ and new emergent infections such as Zika fever. For this reason, there is an increasing global interest in the study of infectious diseases and its link with climate variability.^6^ ^,^ ^7^ A first step in this direction should be to understand whether inter-annual climate oscillations have significant influence on the occurrence of disease outbreaks. However, at present there are scarce studies linking atmospheric oscillations with seasonality and frequency of infectious diseases affecting humans.^8^ ^,^ ^9^

The El-Niño South Oscillation (ENSO) is the major climate pattern taking place in the Pacific Ocean showing seesaws between El Niño (warm) and La Niña (cold) episodes, at intervals of two-seven years. This pattern includes both atmospheric and oceanographic variability. The ENSO is related to the southern oscillation index (SOI), an atmospheric oscillation whose periods of negative values, if prolonged over time, coincide with abnormally warm ocean waters across the eastern tropical Pacific, which is typical of El Niño episodes; instead, long positive periods are related to La Niña (i.e. cold water temperatures). SOI is measured as the difference of air pressure between Tahiti and Darwin station. ENSO has been associated with increases in the occurrence of skin diseases, as well as with infectious diseases such as dengue, leishmaniosis and Chagas.^10^ On the other hand, SOI has shown positive incidence on malaria in five South African countries, which is positively associated to La Niña and negatively correlated to El Niño.^11^

In the same way, the Antarctic oscillation (AAO), an atmospheric low-frequency variability consisting on a large scale change in atmospheric pressure between the Antarctic region and the southern mid-latitudes, is strongly tele-connected to ENSO during the austral summer season peak.^12^ The presence of AAO could explain the highest populations of the long-tail rice rat (*Oligoryzomys longicaudatus*), which is the main Hantavirus reservoir in southern Chile;^13^ but up to now this index has not been directly associated to the development of any disease.

The Chagas disease was described in 1909 by Carlos Chagas, and is caused by the flagellate protozoan *Trypanosoma cruzi*. This parasite is transmitted primarily by blood-sucking triatomine vectors.^14^ The Chagas disease affects at least 21 countries. In America, it extends from the southern states of the USA to Argentina and Chile, and is considered one of the biggest concerns in public health in Latin America.^14^ ^,^ ^15^ ^,^ ^16^. Its last outbreak caused 28,000 new cases per year, with an estimated 15-16 million people infected and 75-90 million exposed to infection.^17^ In Chile, the main vectors involved in the transmission of *T. cruzi* are arthropods of the Triatominae subfamily (Insecta, Hemiptera, Reduviidae): domestic *Triatoma infestans*, which is known by the name of “vinchuca” and wild species of the genus *Mepraia*.^16^ Moreover, another main transmission way is through of vectors by fecal-urine way, however, oral way also has been described among others transmission form.^18^ ^,^ ^19^ ^,^ ^20^ ^,^ ^21^

The Chagas disease is more common in rural and peri-urban areas.^16^ The report of new cases of Chagas disease has become obligatory in Chile from 1990. Since the year 2000, the interruption of vector transmission of Chagas disease was declared, but the vector is still present, and continues vertical transmission which is coupled to a huge cohort of patients in the indeterminate chronic phase.^20^ ^,^ ^21^

The available data show an average of 2.95 cases per 100 thousand habitants until 2008; for 2009, the number of cases increased until 6.79 reports per 100 thousand habitants, and a new 70% increase took place in 2011.^22^ However, the increase has not been associated to meteorological changes or to higher exposition to the vector. These reports have been associated instead to diagnostic-technique improvements at health centers, as well as to an increase in the proportion of cases in older-age groups (which had remained asymptomatic during 10 to 30 years).

In order to get understand outbreaks of Chagas disease, we search for possible relationships between the frequency of cases in the Chilean population and two atmospheric oscillations in the Southern Hemisphere: SOI and AAO, during the available years with official data.

## MATERIALS AND METHODS

We explored trends in the Chagas disease frequency per year (ChDF) (i.e. number of sick people) in Chile. Because the number of migrant people born outside from Chile increasing significantively between 2014 and 2018^20^ (https://www.abc.es/internacional/abci-chile-pais-americano-mayor-aumento-inmigrantes-201806190456_noticia.html), which could distort the data (since people born outside of Chile could have been infected with Chagas in distant places), we used for the analysis two different periods from data available official data: (i) 2001 to 2014, (ii) 2001 to 2017. In relation to the monthly annual average of two atmospheric oscillations: AAO, downloaded from the website of the National Weather Service (http://www.cpc.ncep.noaa.gov/products/precip/CWlink/daily_ao_index/aao/aao.shtml), and the SOI, downloaded from the website of the National Oceanic and Atmospheric Administration (http://www.cpc.ncep.noaa.gov/data/indices/soi) (Table).

We hypothesised that gaps could be produced between the possible effect of atmospheric oscillation and Chagas disease, as a consequence of a possible cascade of events. For this reason, we included a one-year time lag in the atmospheric oscillations. So, we also used as predictor variables the SOI and AAO values recorded one year before [Southern oscillation index in previous year (SOIpy), and Antarctic oscillation in previous year (AAOpy) thereafter] (Table).

**TABLE:** Data used for the study

Year	ChFD	SOI	SOIpy	AAOO	AAOOpy
2001	685	0.4	1.392	0.353	-0.1204
2002	537	-0.525	0.4	-0.6018	0.353
2003	476	-0.15	-0.525	-0.223	-0.6018
2004	516	-0.425	-0.15	0.1886	-0.223
2005	538	-0.3083	-0.425	-0.197	0.189
2006	539	0.0167	-0.3083	0.257	-0.197
2007	436	0.433	0.0167	-0.468	0.257
2008	592	1.883	0.433	0.671	-0.468
2009	1152	0.275	1.883	-0.191	0.671
2010	1170	1.525	0.275	0.786	-0.193
2011	1991	2.3083	1.525	-0.0547	0.786
2012	1339	0.225	2.3083	0.133	-0.0547
2013	958	0.75	0.225	0.0317	0.133
2014	1018	-0.22	0.75	0.08	0.03
2015	1264	-1.33	-0.22	0.71	0.08
2016	1370	-0.19	-1.33	0.57	0.71
2017	1507	0.36	-0.19	0.45	0.57

ChDF: Chagas diseases frequency; SOI: annual average for the monthly South oscillation index; SOIpy: annual average for the monthly South oscillation index previous year; AAOO: annual average for the Antarctic oscillation index; AAOOpy: annual average for the Antarctic oscillation index previous year.

*Data analysis* - In a first step, we analysed the time series for each variable. We searched for temporal autocorrelation and cyclicity in the time series using spectral analysis, to identify periodicity. Time-autocorrelation and spectral analysis was performed with the software PAST (available from website: http://folk.uio.no/ohammer/past/).^23^ ^,^ ^24^

Pearson correlation was used to measure covariations between ChDF and the annual average of different monthly Southern Pacific atmospheric oscillations (i.e. SOI, SOIpy, AAO, and AAOpy, where “py” denotes values recorded the previous year). The relationship between ChDF as a dependent variable and the annual average of different monthly Southern Pacific atmospheric oscillations as independent variables was determined by stepwise linear regression. This regression is based on achieving the highest F-value while minimising collinearity of variables in the final model. The normality of the distribution of the variables was tested using the Kolmogorov-Smirnov test for one sample.^25^

Due to the fact that the official value on number of Chagas patients includes both new cases and chronic cases, the frequency anomaly was used (that is, the observed frequency of one year, minus the average of the period). Thus we obtained values above the average (positive), as below the average (negative). Positive and negative values were transformed into 1 (for positives) and 0 (for negatives). This new binary descriptor was used as the dependent variable to test whether any of the climatic oscillations increased the probability of observing a frequency above the average. This test was performed using forward/backward-stepwise binary logistic regressions.

Model coefficients were evaluated using the omnibus test and Hosmer and Lemeshow test, both follows a Chi-square distribution.^26^ Moreover, the discrimination capacity of the model was evaluated with the area under the receiving operating characteristic curve (AUC). The relative importance of each variable within the model was assessed using the Wald test.^26^

The relation between the different climatic indices and ChDF can be also analysed in terms of the accumulated values.^27^ Annual values were transformed into anomalies by subtracting the mean value calculated over the whole period 2001-2014. The accumulated values corresponding to specific years were then calculated as the sum of the anomalies of the previous years (e.g. the accumulated values corresponding to 2010 were calculated as the sum of the anomalies for the period 2001-2014), according to the expression:

$\sum_{i=m}^{n}Annual\;valuei-Mean\;period$

where n is the reference year, the annual value of the variable is referred to a particular year (i), and the mean period is the average of the variable values for the whole studied period (i.e. since the initial year m = 2001 to the last year n = 2014).

## RESULTS

*Period 2001 to 2014, before the increase in migration* - We did not observe temporal autocorrelation or time trend in the study variables. Instead, we detected a significant correlation between ChDF and SOI (r = 0.6, p = 0.024, N = 14), SOIpy (r = 0.704, p = 0.005, N = 14) and AAOOpy (r = 0.551, p = 0.041, N = 14).

**Fig. 1:**
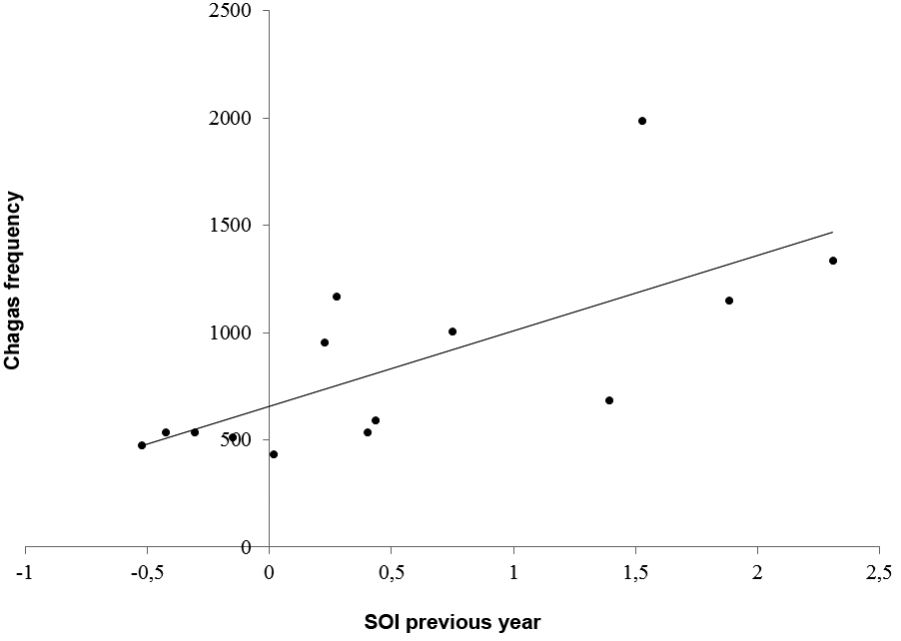
relationship between South oscillation index (SOI) between Chagas frequency from Chile for the study period.

A positive significant relationship between ChDF and SOIpy was also observed through the following linear equation (F = 11.82, p = 0.005, R^2 = 0.496, Durbin-Watson = 1.284) (Fig. 1):

ChDF = 657.018+ SOIpy*350.738

For binary anomalies, we found a statistically significant and positive logistic regression with SOIpy. The model’s goodness-of-fit was significant according to the Hosmer and Lemeshow test (Chi-squared = 6.768, df = 8, p = 0.562), and its discrimination capacity was good (AUC = 0.854). The logit function (y) of the logistic regression was: 

Y = 1.357 + 1.921*SOIpy

**Fig. 2:**
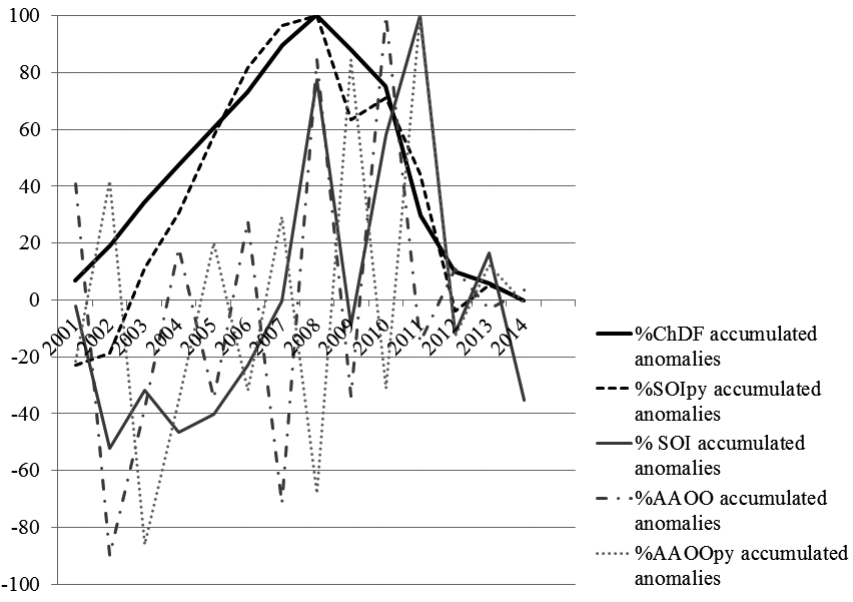
accumulated anomalies trend in percentages for the studied period (2001-2014), for: Chagas diseases frequency (ChDF), annual average for the monthly South oscillation index previous year (SOIpy), annual average for the monthly South oscillation index (SOI), annual average for the Antarctic oscillation index (AAOO), and annual average for the Antarctic oscillation index previous year (AAOOpy).

In both models, positive SOIpy showed to be an important independent variable to explain the frequency of Chagas disease. The average frequency in 2003, 2004, 2005 and 2006, one year after average monthly SOI showed negative values, was 517; whereas the average frequency in 2007, one year after positive values (= 0.0167), was 987. We observed a similar trend in the analysis of accumulated values (Fig. 2).

*Period 2001 to 2017, after the increase in migration* - We also observed a significant correlation between ChDF and AAOpy (r = 0.624, p = 0.0007, N = 17). A positive significant relationship between ChDF and AAOpy was also observed through the following linear equation:

ChDF = 867,713 + AAOpy*695.295 (F = 9.573, p = 0.007, R^2 = 0.35)

## DISCUSSION

On the one hand, Chile implemented a vector control program, which resulted in the elimination of *T. infestans* colonies from domestic spaces, interrupting vectorial transmission to humans in 1999.^28^ However, data show increasing incidence of Chagas’ disease.^29^ Moreover, sylvatic vector populations are present in rural and metropolitan areas, infecting sylvatic and synanthropic mammals species.^30^ On the other hand, the number of migrant people born outside from Chile increasing significantively between 2014 and 2018 (https://www.abc.es/internacional/abci-chile-pais-americano-mayor-aumento-inmigrantes-201806190456_noticia.html),^14^ for this reason the increasing in the Chagas frequency from these years should be used with caution. Nevertheless, for two different periods analysed here, we observed significant climatic oscillation correlation.

In the current study we found that La Niña phases (related with positive SOI) in previous year could favor the increase of Chagas disease cases. This result could be due to an effect on the enlargement of vector populations involved in the transmission of *T. cruzi* in Chile. In contrast, *T. cruzi* infections in native rodents from Chile, where a higher prevalence of infection on mammals per unit of area was associated during El Niño events.^31^ This apparent contradiction in the results of both studies could be related. Thus, La Niña phases are continuing with El Niño phases. Therefore, if during La Niña phases with one year of gap favor the increasing to Chagas disease cases, in consecutive years, during El Niño phases, it is possible to observe more cases.

The present results lack of biological explanation for the associations Chagas and atmospheric indices. In this context, present finding, due be considered as first approximation to this issue. Moreover, further studies should provide more evidence in this regard. In this sense, the main weakness of the present study is the short series of data studied.

Menu et al.^32^ performed a mathematical model suggesting the existence of dynamic interactions between the evolution and epidemiology of Chagas vector as responses to global climatic change.^32^ In addition, it has been proposed that changes in the frequency of Chagas disease in Argentina and Venezuela, specifically in the rural populations, could be highly affected for climatic projections.^33^ Climate variability over South America, specifically in Uruguay and Argentina, has shown to influence the development of vectors including those of the Chagas disease.^34^ On the other hand, it is expected that climatic change alter El Niño-La Niña pattern.^35^ In this context, the present results due be considered, because it is possible observed a increasing of Chagas disease in all South American region.

The ChDF in Chile was found to depend mainly on SOIpy, whose values can be determined one year in advance. Therefore, it is possible to partially forecast annual frequency patterns. This could have important applications in public health strategies and for allocating resources for the management of the disease.
